# Executive functions in developmental dyslexia

**DOI:** 10.3389/fnhum.2014.00120

**Published:** 2014-03-07

**Authors:** Pamela Varvara, Cristiana Varuzza, Anna C. P. Sorrentino, Stefano Vicari, Deny Menghini

**Affiliations:** ^1^Neuroscience Department, Children's Hospital Bambino GesùRome, Italy; ^2^Psychology Department, Libera Università Maria Ss. AssuntaRome, Italy

**Keywords:** developmental disabilities, working memory, central executive system, attention, phonological processing

## Abstract

The present study was aimed at investigating different aspects of Executive Functions (EF) in children with Developmental Dyslexia (DD). A neuropsychological battery tapping verbal fluency, spoonerism, attention, verbal shifting, short-term and working memory was used to assess 60 children with DD and 65 with typical reading (TR) abilities. Compared to their controls, children with DD showed deficits in several EF domains such as verbal categorical and phonological fluency, visual-spatial and auditory attention, spoonerism, verbal and visual short-term memory, and verbal working memory. Moreover, exploring predictive relationships between EF measures and reading, we found that spoonerism abilities better explained word and non-word reading deficits. Although to a lesser extent, auditory and visual-spatial attention also explained the increased percentage of variance related to reading deficit. EF deficits found in DD are interpreted as an expression of a deficient functioning of the Central Executive System and are discussed in the context of the recent temporal sampling theory.

## Introduction

Executive functions (EF) are a set of high cognitive abilities that control and regulate other functions and behaviors (Welsh et al., 1991). They may involve abilities such as selectively processing information in the environment, retaining task-relevant information in an accessible state over time, making a plan by selecting a sequence of actions to achieve a goal, inhibiting a verbal or motor response, successfully adapting responses to changes in situations and environments, problem solving and self monitoring (Welsh et al., 1991; Pennington and Ozonoff, 1996; Friedman et al., 2006).

EF deficits have been described in several developmental disorders. Attention, working memory, inhibitory control, and flexible thinking deficits have been documented in individuals with attention and hyperactivity disorders (Sergeant et al., 2002; Corbett et al., 2009; Abad-Mas et al., 2011; Pani et al., 2013); inhibition of responses and planning deficits have been described in children with autism (Rinehart et al., 2001; Hill, 2004; Kenworthy et al., 2005; Robinson et al., 2009); planning and memory deficits have been reported in fetal alcohol spectrum disorder (Rasmussen, 2005; Green et al., 2009; Pei et al., 2011). Deficits in a set of EF have also been documented in genetic syndromes associated with intellectual disability (Costanzo et al., 2013). Moreover, a recent meta-analysis (Booth et al., 2010) has highlighted that children with reading disability have difficulties in several EF, including maintaining relevant information in WM, inhibition of irrelevant information, and accessing material in long-term memory.

Indeed, deficits in WM have been mainly investigated using span tasks and are considered one of the major defining characteristics of Developmental Dyslexia (DD) (Willcutt et al., 2005; Swanson et al., 2009, 2010; Bacon et al., 2013). Both verbal and visual-spatial components of WM have been found impaired in children (Poblano et al., 2000; Brosnan et al., 2002; Helland and Asbjørnsen, 2004; Martinussen and Tannock, 2006; Smith-Spark and Fisk, 2007; Menghini et al., 2011) and adults (Smith-Spark et al., 2003; Alloway and Alloway, 2013) with DD. In the light of Baddeley's model, the reduced performance of dyslexics in memory span tasks involving both verbal and visual-spatial modalities may be interpreted as an expression of deficient functioning of the Central Executive component of WM. The tripartite WM model (Baddeley and Hitch, 1974; Baddeley and Logie, 1999; Baddeley, 2000, 2001) includes a Central Executive System (CES) responding to different cognitive functions (e.g., attention, active-inhibition, planning, updating, maintenance and integration of information) and two peripheral slave systems devoted to the temporary storing and rehearsing of information pertaining to a single modality (visual-spatial sketchpad and articulatory loop). The CES coordinates the two slave systems, integrating their storage capacities and making available attentional resources for online processing of incoming information. It has been proposed that the CES may be analogous to the Supervisory Attentional System (SAS) described by Shallice and colleagues (Norman and Shallice, 1986; Shallice and Burgess, 1993). Indeed, the SAS is defined as a conscious control mechanism that resolves interference and allows attentional control over action. CES and SAS not only have a critical control function in WM, but are also involved in several processes requiring higher-order cognitive control. Accordingly, Kane and Engle (2002) define controlled attention as an executive control capability that can effectively maintain stimulus, goal, or context information in an active, easily accessible state in the face of interference, effectively inhibiting goal-irrelevant stimuli or responses (Kane et al., 2001).

Deficits in visual-spatial attention in DD have also been found using tasks which evaluate orientation, focusing, shifting attention and visual search (Helland and Asbjørnsen, 2000; Altemeier et al., 2008; Menghini et al., 2010). Deficits in auditory attention have also been found using discrimination speech tasks (e.g., Casco and Prunetti, 1996; Facoetti et al., 2000; Buchholz and McKone, 2004; Valdois et al., 2004; Dufor et al., 2007). Hari and Renvall (2001) and Laasonen et al. (2012) have suggested that children with DD may suffer from sluggish attentional shifting (i.e., engaging and disengaging attention) as revealed by tasks assessing sustained and divided attention, auditory saltation illusion, auditory pitch streaming sequence, and attentional blink.

Furthermore, studies in DD have documented deficits in categorical fluency, planning, monitoring and revising during problem solving, and response shifting using the flexibility task, the Go/No-go task, and the Tower of London task (e.g., Condor et al., 1995; Mati-Zissi and Zafiropoulou, 2001; Reiter et al., 2005; Swanson et al., 2006). Other authors have demonstrated that DD children were impaired in inhibition tasks such as the Stroop Test (Everatt et al., 1997; Brosnan et al., 2002; Reiter et al., 2005).

With few exceptions (Reiter et al., 2005; Willcutt et al., 2005; Swanson et al., 2006), the above-mentioned studies investigated each EF separately in DD.

However, in the last few years, a multifactorial deficit has been found, supporting the view that neurocognitive developmental dysfunctions in DD may not be limited to the linguistic brain areas, but may also involve a more multifocal cortical system (Pennington, 2006).

Recent functional neuroimaging findings provide evidence of a neurobiological signature for dyslexia, including two crucial posterior systems, one in the occipito-temporal regions and one in the parieto-temporal regions, as well as an anterior system, mainly located in the inferior frontal gyrus (Shaywitz et al., 2008).

The anterior system is implicated in the output of phonological and articulatory aspects, verbal working memory, and the semantic aspects of word reading (Price, 2000; Jobard et al., 2003; Gabrieli, 2009). It was recently suggested (Hoeft et al., 2007; Graves et al., 2010) that in both expert readers and dyslexics the contribution of this system to reading is primarily due to the attention, working memory and executive processes required by reading than to orthographical-phonological mapping *per se*.

The occipito-temporal system has a particularly important role in skilled, fluent reading (i.e., rapid and automatic identification of words) and encompasses the fusiform gyrus (Visual Word Form Area) and the middle and inferior temporal gyrus (Cohen et al., 2000; Vinckier et al., 2007). The parieto-temporal system includes the angular and the supramarginal gyri, and portions of the temporal lobe, such as the left superior temporal gyrus. This system is responsible for cross-modal relation of auditory and visual processes during reading and for the integration of letters and sounds.

To overcome the limitations of previous studies which evaluated each EF separately, the aim of the present study was to simultaneously test different EF domains in the same group of children with DD using numerous tasks.

Children with DD were then compared to children with typical reading (TR) abilities using a neuropsychological battery tapping several EF such as verbal phonological and categorical fluency, spoonerism abilities, visual-spatial and auditory attention, verbal, visual and spatial short-term memory, verbal WM, and visual shifting. To better understand the potential role of EF in reading, the relationships between EF measures and reading abilities were also explored.

## Materials and methods

### Participants

The study included 60 children and adolescents with DD (27 females) and 65 children with TR abilities (28 females) matched for chronological and mental age.

The chronological age range was 8–17 years for the DD group (mean 11.4 ± 1.9 *SD*) and 8 to 16 years for TR participants (mean 11.9 ± 1.6 *SD*). The diagnosis of DD was made when word or non-word reading speed and/or accuracy level was at least 2 standard deviations below the mean value for the scholar level. In a non-verbal intelligence test Colored Progressive Matrices (CPM; Raven, 1994) all DD participants had a performance level in the normal range (above 10th percentile; mean 29.28 ± 4 *SD*).

The presence of Attention Deficit Hyperactivity Disorder was excluded by means of DSM-IV recommendations (American Psychiatric Association, 2000) and confirmed by behavioral rating scales filled out by parents (Cornoldi et al., 1996; Re and Cornoldi, 2009). Furthermore, none of the children with DD underwent any intensive or specific reading training.

Criteria for inclusion in the TR group were: (i) no reading delay in word and non-word reading tests; (ii) normal or corrected visual acuity; (iii) no Attention Deficit Hyperactivity Disorder diagnosis. In the above-mentioned non-verbal intelligence test (Raven, 1994), TR children had a performance level in the normal range (above 10th percentile; mean 29.83 ± 3.53 *SD*).

### Design and materials

Children with DD were evaluated in three testing sessions carried out on three different days at the Department of Child and Adolescent Psychiatry of the Bambino Gesù Children's Hospital in Rome. TR children were assessed in three testing sessions in a local primary school. All tasks included a practice phase during which the experimenter explained the task instructions. Tasks were administered in a pseudorandom manner (IQ and reading abilities were always assessed before testing began). All the children's parents gave written informed consent after an extensive description of the research study. The neuropsychological battery involved IQ, reading abilities and EF tasks.

### General intelligence

Non-verbal intelligence was measured using the CPM (Raven, 1994), which evaluate the ability to form perceptual relations and to reason by analogy, irrespective of language and formal schooling.

### Reading abilities

Reading abilities were assessed using two subtests of a battery for the diagnosis of DD (Sartori et al., 1995). Participants were asked to read aloud lists of words and non-words. Speed (in seconds) and errors (each incorrect word or non-word was calculated as one error) were computed for each task. Also, inefficiency reading indexes were considered, calculating the ratio between reading speed and accuracy rate (number of words/non-words correctly read over the total number of words/non-words) for both word and non-word reading tasks.

### EF tasks

#### Verbal categorical fluency

The Category fluency test (Vicari, 2007) was used to measure verbal fluency. Participants were asked to generate words in a particular category (e.g., animals, clothes, fruits, toys). Deviations from the test rules, including repetitions (perseveration errors) and words not identifiable as an example of the category, were considered errors. All words generated by the participants were recorded by the examiner, and the number of valid responses produced during the time limit (60 s) was calculated (excluding repetitions and errors). The score is calculated as the sum of the number of words generated for each category and the total number of responses.

#### Verbal phonological fluency

The Phonological Fluency Test (Marotta et al., 2008) was used to evaluate the ability to recall several items using a phonological input. In this task, the child was asked to verbalize as many words as possible beginning with a given phoneme (F, A, and S) within 60 s for each of the three trials. The global score is the sum of the correct responses for the three trials.

#### Spoonerism

The Spoonerism Task (Marotta et al., 2008) was used to evaluate phonological awareness. The examiner pronounced two words aloud and the participant had to swap the initial phonemes to form two new real words. The child was asked to transpose the beginning sounds of the two words as quickly as possible (time limit to complete a single trial: 60 s; number of trials: 15). The score is the number of correct answers (maximum score: 30) and the time taken to complete the entire test (15 trials).

#### Visual-spatial attention

Selective visual-spatial attention was evaluated using the Map Mission. In this subtest of the Test of Everyday Attention for Children (Manly et al., 2001), a color-printed A3-laminated city map was presented. Eighty targets representing restaurants (i.e., small knife and fork symbols) were randomly distributed across the map. Distracting symbols of the same size were also present. Participants used a pen to circle as many targets as possible in 60 s. The performance score is the number of target symbols correctly marked by the participants (maximum score: 80).

#### Auditory attention

Sustained auditory attention was investigated using the Code Transmission task. In this task, which was also a subtest of the Test of Everyday Attention for Children (Manly et al., 2001), participants were asked to monitor a stream of monotonous digits (presented at a rate of one every 2 s) for the occurrence of a particular target sequence (e.g., 5, 5) and then to report the digit that occurred immediately before the target sequence. After a practice sequence to ensure comprehension, 40 targets were presented. The number of targets correctly detected is recorded as the measure of performance accuracy (maximum score: 40).

#### Verbal short term and working memory

To assess verbal short term memory and WM a verbal span task from an extensive memory battery was used (Vicari, 2007). The task consists of a list of eight, two-syllable low frequency words. In the first block, the examiner read aloud two words at a rate of one item per second. The participants were required to repeat the two words in the same order. Then, four additional strings of two words were presented. If the child was successful in at least three of the five sequences, a sequence one word longer was presented. If the child failed (less than three correct answers in a block), the task was discontinued. The same procedure was used for sequences of increasing length (up to a maximum of seven words). The score is computed by assigning 0.5 to each sequence of items correctly reproduced by the participant (maximum score: 17.5).

Moreover, a non-word repetition task (Vicari, 2007) was used to assess verbal WM. The number of non-words correctly repeated is recorded as a measure of performance accuracy (maximum score: 40).

#### Spatial short term and working memory

In the visual-spatial span task (Vicari, 2007), the material consisted of a non-verbalizable geometric shape that appears for 2 s in one of seven possible positions on the computer screen. Then, two empty cells were presented in the same spatial positions as before and the child had to indicate the order in which the stimuli appeared. If the child was successful in at least three of five two-position sequences, a sequence one block longer was presented. Also in this case, the same testing procedure was used for sequences of increasing length (up to a maximum of seven spatial positions). The score was computed by assigning 0.5 to each sequence of items correctly reproduced by the participant (maximum score: 17.5).

#### Visual short term and working memory

A similar procedure was used for the visual span task (Vicari, 2007). In this case, the experimental material consisted of seven complex geometric figures depicted in high contrast colors. Two figures were presented, one at time, for 2 s at the center of the computer screen. After the disappearance of the second figure, the two figures were presented aligned in the center of the screen in a random position and the participant was asked to indicate the order in which they appeared. Also in this case, if the child was successful in at least three of the five trials, a sequence one figure longer was presented and the task continued until a maximum of seven figures have been presented. The score is computed by assigning 0.5 to each sequence of items correctly reproduced by the participant (maximum score: 17.5).

#### Visual shifting

The Wisconsin Card Sorting Test (WCST; Heaton et al., 1993) is a neuropsychological test of set shifting and also involves cognitive flexibility function. The WCST consists of four stimulus cards and 128 response cards which differ in color (red, green, blue and yellow), shape (circle, star, cross, and star) and number (one, two, three, and four). The stimulus cards are one red triangle, two green stars, three yellow crosses, and four blue circles. The child was given the response cards and instructed to place each consecutive card under one of the stimulus cards, according to which he/she considered correct. After each sort, the child was informed whether he/she was correct. The first sorting category was color, and after 10 consecutive correct sorts, the category changed to form, without forewarning, and then accordingly to number. Errors in shifting from one category to another and perseveration errors are registered as scores.

### Statistical analysis

Data were analyzed using the Statistical Program for Windows, Version 8.0 (StatSoft, Inc., Tulsa, OK, USA). Participants' performances were transformed into z-scores. Since the assumptions of normality were not met, comparisons between DD and TR children on EF measures were carried out by means of the Kruskal-Wallis non parametric test with Group as independent between-subjects factor. To correct for multiple comparisons, the level of significance was set at *p* = 0.005 using Bonferroni correction (10 comparisons).

Moreover, multiple regression analyses were computed to explore the linear relationship between EF measures (predictor variables) and reading abilities (criterion variable), using the stepwise method.

## Results

DD children obtained significantly lower scores (*p* always ≤ 0.005) than TR children in phonological and categorical fluency, spoonerism abilities, visual-spatial and auditory attention, verbal and visual short-term memory, and verbal WM. For the level of significance adopted (*p* = 0.005), the comparisons of spatial short-term memory and shifting abilities showed no significant results (see Table 1).

**Table 1:** **Performances of children with developmental dyslexia (DD) and with typical reading (TR) in executive functions tasks (after back-transformation to original measure units)**.

**Measure**	**DD Mean (*SD*)**	**TR Mean (*SD*)**	***X*^2^**	***p***
Phonological fluency (correct responses)	21 (8.26)	25.97 (6.61)	9.03	0.0027
Categorical fluency (correct responses)	43.40 (8.48)	50.97 (7.35)	19.74	<0.0001
Spoonerism (speed in seconds)	413.55 (162.81)	123.69 (76.60)	91.81	<0.0001
Visual-spatial attention (correct responses)	37.15 (10.43)	43.91 (11.69)	7.81	0.005
Auditory attention (correct responses)	34.05 (5.13)	38.22 (2.15)	17.49	<0.0001
Verbal short-term memory (span score)	3.55 (0.62)	4.08 (0.54)	9.76	0.0018
Visual short-term memory (span score)	3.10 (0.48)	3.68 (0.85)	11.20	0.0008
Spatial short-term memory (span score)	4.80 (0.80)	4.94 (0.79)	7.42	0.0064
Verbal working memory (correct responses)	31 (3.54)	37.18 (1.93)	58.09	<0.0001
Non-verbal shifting (errors)	73.18 (13.78)	75.51 (9.28)	0.01	1.0

To determine predictive relationships between EF measures (predictor variables) and inefficiency reading indexes (criterion variables), multiple regression analyses were computed using the stepwise method. Thus, each EF measure was entered in sequence as predictor variable and its value assessed to determine its contribution to the success of the model; variables that did not significantly contribute were automatically removed.

Overall, the first regression model, which included only spoonerism abilities (speed in seconds), accounted for 49.2% of the variance (*R*^2^ change = 0.492) in words reading inefficiency index. The inclusion of auditory attention resulted in an additional 4.2% of the variance being explained (*R*^2^ change = 0.042). The addition of visual-spatial attention explained a further 2.8% of the variance (*R*^2^ change = 0.028).

Similarly to non-words reading inefficiency, the first regression model included only spoonerism abilities, accounting for 49.2% of the variance (*R*^2^ change = 0.492). The inclusion of auditory attention explained an additional 3% of the variance (*R*^2^ change = 0.03), and the inclusion of visual-spatial attention accounted for a further 2% of the variance (*R*^2^ change = 0.02). Table 2 summarizes multiple regression analyses for words and non-words reading inefficiency index.

**Table 2:** **Multiple regression analysis predicting words and non-words reading inefficiency (only significant relationships are shown)**.

	**Words**				**Non-words**			
**Predictor variable**	**β**	***t* for β**	***p***	**Adjusted *R*^2^**	**β**	***t* for β**	***p***	**Adjusted *R*^2^**
Spoonerism (speed in seconds)	−0.55	−7.956	<0.001	0.488	−0.575	−8.122	<0.001	0.488
Auditory attention (correct responses)	−0.204	−3.052	0.003	0.527	−0.171	−2.493	0.014	0.514
Visual-spatial attention (speed in seconds)	−0.181	−2.798	0.006	0.552	−0.152	−2.295	0.023	0.53

## Discussion

This study was aimed at testing simultaneously different EF domains in a group of DD children. Deficits in several aspects of EF such as spoonerism, verbal categorical and phonological fluency, visual-spatial and auditory attention, verbal and visual short-term memory, and verbal WM have been found. However, spatial short-term memory and visual shifting abilities were preserved.

Consistent with previous studies, we observed a deficit in verbal phonological fluency (Goswami, 2000; Snowling, 2000; Ramus, 2003; Ramus et al., 2013) and in verbal categorical fluency (Snowling, 2000; Ramus et al., 2003; Reiter et al., 2005). These tasks engage complex cognitive mechanisms, such as WM, self-monitoring, and flexible thinking (Troyer et al., 1998; Schwartz et al., 2003), and in addition require rapid access to words and strategic search through lexical/phonologic and conceptual/semantic memory (Baldo and Dronkers, 2006). Due to the several cognitive mechanisms involved, a large cortical network is required, including the dorsolateral prefrontal cortex and word associative fronto-temporal regions (Frith et al., 1991; Friston et al., 1991; Cilia et al., 2007; Kalbe et al., 2009).

We also found in children with DD a lower performance in the spoonerism task, which is generally used to assess phonological awareness. However, to interpret this result properly it is necessary to focus on the extra task demands and not only on phonological abilities (Wimmer et al., 2000). The child is first asked to segment the word into two parts, the first phoneme (onset) and the remainder of the word (rime), and temporally store the onset and rime of the first word; the child repeats this segmentation for a second word, and then blends the first onset with the second rime, and the second onset with the first rime. Therefore, the spoonerism task requires blending as well as segmentation skills, but also involves short-term and WM abilities, close monitoring of the phonological manipulation, and inhibitory processes. These complex demands could explain the deficits in tasks such as spoonerism usually found in dyslexic children (Jeffries and Everatt, 2004; Berninger et al., 2008; Kibby and Cohen, 2008; Menghini et al., 2011). It is worth noting that the spoonerism task, which involves all of these functions, has an important predictive role for both global and analytic reading skills.

Moreover, our results concerning attention support the hypothesis that both phonological processing and non-verbal processing are impaired in DD. Indeed, significant differences between DD and TR children were found in auditory and visual-spatial attention domains. Although to a lesser extent, our results support the concept that auditory and visual-spatial attention explains an increased percentage of the variance related to reading disorders. A contribution of attention to reading, independent of the sensory modality considered, is then confirmed. Previous studies generally evaluated visual and auditory attention separately. Concerning visual attention, our results are consistent with reports of difficulties in rapidly focusing visual attention observed in individuals with DD (e.g., Brannan and Williams, 1987) as well as deficits in automatic control of visual attention (Facoetti et al., 2000, 2001, 2003; Hari and Renvall, 2001). Moreover, it was found that visual-spatial attention in preschoolers specifically predicts future reading acquisition (Franceschini et al., 2012), suggesting new approaches for early identification and efficient prevention of DD.

With respect to auditory attention, studies have clearly demonstrated auditory attentional deficits in DD (Asbjornsen and Bryden, 1998; Facoetti et al., 2000; Goswami, 2000; Ramus, 2003; Tallal, 2004; Ramus et al., 2013) which have been interpreted not only as a deficit in speech-sound perception (Cunningham et al., 2001) and in processing rapid sound sequences (Helenius et al., 1999), but also as a problem in shifting and focusing auditory attention (Renvall and Hari, 2002). The few studies assessing DD children on both modalities have documented multimodal (i.e., visual and auditory) attention deficits (Facoetti et al., 2000, 2003; Buchholz and McKone, 2004; Valdois et al., 2004; Dufor et al., 2007).

Furthermore, there is some evidence that in TR a visual dorsal pathway provides a mechanism for the early, preattentive visual analysis of words (Vidyasagar, 1999, 2001; Pammer et al., 2006; Vidyasagar and Pammer, 2010). An adequate parsing of a stream of text into grapheme guides the grapheme-phoneme correspondence and the sensory integration of visual signals. For the proper identification of the graphemes and the subsequent matching of graphemes with phonemes, an attentional mechanism is required. The multimodal integration of visual and auditory inputs mediates the synthesis of orthographic and phonological information (Pammer et al., 2006).

In our group of dyslexic children a general multimodal (verbal and visual) deficit in short-term memory and WM has also been found. The results are at variance with the theory that dyslexic children have an isolated verbal short-term deficit, possibly secondary to a deficit of phonological processing or the expression of a dysfunctional articulatory loop (Poblano et al., 2000; Willcutt et al., 2001; Jeffries and Everatt, 2004; Kibby et al., 2004). By contrast, our results concord with data showing deficits in individuals with DD in the temporary storage of visual-spatial as well as verbal material (Poblano et al., 2000; Brosnan et al., 2002; Helland and Asbjørnsen, 2004; Martinussen and Tannock, 2006; Smith-Spark and Fisk, 2007; Menghini et al., 2011). Our findings on WM can be interpreted as a deficient functioning of CES or SAS (Baddeley and Hitch, 1974; Norman and Shallice, 1986; Shallice and Burgess, 1993; Baddeley, 2000, 2001). Indeed, consistent with the model of WM, the failure of the CES to supervise the activity of both peripheral slave systems could fully account for poor performances in tasks involving different modalities as found in our dyslexic children.

Considering the several processes involved in the EF tasks adopted (e.g., attention, active-inhibition, temporary storage, maintenance, update and integration of information from several domain-specific codes) and their cross-modalities nature, deficits found in our dyslexic children could be the expression of a deficient functioning of CES/SAS. Therefore, we propose that a more global deficit in higher-order cognitive mechanisms might be a crucial feature of DD.

Additional support to this hypothesis is given by our findings which show that some EF tasks, primarily spoonerism but also auditory and visual-spatial attention, are strictly related to reading deficits in DD children. These data could confirm the contribution of the executive attention and domain-general attention control abilities (e.g., CES/SAS) to reading, irrespective of the sensory modality.

However, an additional hypothesis cannot be excluded. During recent years it has been proposed that DD arises from an abnormal auditory sampling (Goswami, 2011). Since cortical oscillations have been implicated in several aspects of human cognition, it has been assumed that the abnormal phonological processing observed in DD reflects a deficit in temporal sampling of speech by neuroelectric oscillation that encodes incoming information at different frequencies (Goswami, 2011). Indeed, temporal coding via the synchronous activity of oscillating networks of neurons at different frequency bands is crucial in the perceptual processing of speech (Luo and Poeppel, 2007). In dyslexics the auditory sampling might be altered, yielding phonemic representations of an unusual temporal format, with specific consequences for phonological processing and phoneme/grapheme associations (Lehongre et al., 2011).

Similarly, studying high-frequency auditory oscillations associated with verbal WM, Lehongre et al. (2011) found that dyslexics exhibited a supranormal entrainment of bilateral planum temporal to fast temporal modulations in the 50–70 Hz range. It has been hypothesized (Lehongre et al., 2011) that in dyslexics too fast low-gamma oscillations might flood the auditory system with overdetailed spectrotemporal information, thereby saturating theta-based auditory buffer capacity and verbal WM. However, the relationship between neuronal oscillations and non-verbal WM correlates is still an open issue in DD.

Finally, the cellular synchronization of oscillatory responses might be responsible for the above mentioned multimodal integration of visual and auditory inputs which mediates the synthesis of orthographic and phonological information (Gray et al., 1989). Indeed, changes in gamma-band signals have been implicated in cognitive integration of stimuli (Pulvermüller et al., 1997; Ward, 2003; Hermann et al., 2004) and alpha-band activity contributes to specific attentional processes such as attentional selection and filter. Moreover, alpha power is larger over visual cortices when attention is focused on the auditory part of a multimodal auditory-visual stimulus (Klimesch, 2012). So, different frequency domains might interact in terms of cross-frequency coupling of oscillations, with cross-modal knock-on effects. One possibility is that atypical cross-frequency coupling in DD might interfere with the attentional mechanism for the multimodal integration of visual and auditory inputs.

In conclusion, our findings support the hypothesis that DD is a multiple neurocognitive deficit and not solely related to a phonological system dysfunction. The relation of brain oscillation to EF networks remains to be explored and could be useful for the further development of current neurophysiological models of DD.

### Conflict of interest statement

The authors declare that the research was conducted in the absence of any commercial or financial relationships that could be construed as a potential conflict of interest.
